# A Path to Better-Quality mHealth Apps

**DOI:** 10.2196/10414

**Published:** 2018-07-30

**Authors:** Richard S Larson

**Affiliations:** ^1^ School of Medicine University of New Mexico Health Sciences Center Albuquerque, NM United States

**Keywords:** mobile apps, smartphone, software validation, medical device legislation, United States Food and Drug Administration

## Abstract

The rapid growth of mobile health (mHealth) apps has resulted in confusion among health care providers and the public about which products rely on evidence-based medicine. Only a small subset of mHealth apps are regulated by the US Food and Drug Administration. The system similar to that used to accredit and certify laboratory testing under the Clinical Laboratory Improvement Amendment offers a potential model for ensuring basic standards of quality and safety for mHealth apps. With these products expanding into the realm of diagnosis and treatment, physicians and consumers are in a strong position to demand oversight that delivers safe and high-quality mHealth apps.

## Introduction

Since smartphones first went on the market a decade ago, the estimated number of available mobile health (mHealth) apps increased to approximately 325,000 in 2017 [1]. These tools could have a significant positive impact on health and health care. Yet, they also pose novel challenges for patients and clinicians faced with a virtually infinite choice of unproven products.

Doctors struggle with which apps to recommend for patients and patients don’t know which apps may be useful. Physicians must consider the value of an mHealth app before they recommend one since most apps have been created without medical expert involvement or appropriate testing validation [2]. There is a paucity of evidence about the effectiveness of most mHealth apps. One team of US and European researchers went to the iTunes App Store and Google Play in January 2017 in search of apps that can help people cope with anxiety disorders. Of the 52 apps selected, the majority (33/52, 63.5%) offered no information about the intervention. More than two-thirds (35/52, 67.3%) did not offer information about the professional credentials of the app developers or consultants. Only 4% (2/52) offered any information about the efficacy data supporting the apps [3].

Furthermore, apps that provide patient information to physicians must also abide by federal laws protecting personal data and should demonstrate that they are based on best medical practices. Doctors who recommend mHealth apps should be aware that they face potential liability if claims made by app developers are fraudulent [4]. The stakes are rising as new apps and wearable devices come on the market with the ability to gather patient-specific data that can provide clinicians with diagnostic and treatment recommendations. These offer tools that are potentially useful but could affect patients if they rely on false or obsolete medical information [2]. As a result, doctors are wary of recommending mHealth apps. Patients are equally unsure and therefore infrequently get advice about the use of apps from their health care provider.

## Regulatory Efforts

What can patients and physicians do to ensure high-quality mHealth apps? At least four models have evolved for ensuring that organizations who provide products or services to the public meet basic standards of safety and effectiveness. They are regulatory approval by a federal agency, accreditation by an organization with deeming authority under federal law or regulation, voluntary accreditation by a nonprofit organization, and the European Union’s (EU’s) decentralized system driven by its member states. The US Food and Drug Administration (FDA) applies regulatory oversight only to a small subset of mHealth apps. The FDA has indicated that it will regulate only those apps it defines as a medical device and those which potentially pose a risk to patient safety. In a series of nonbinding guidance documents, the FDA has clarified that it will only apply regulatory oversight to apps that “pose a risk to a patient’s safety if the mobile app were to not function as intended” [5]. The FDA also said it intends to regulate those apps that “transform a mobile platform into a regulated medical device” [5] by using display screens, sensors, or other methods. The FDA also reserves “enforcement discretion” for all mHealth apps, meaning it retains the right to regulate what it calls low-risk medical apps [5].

Researchers have defined four broad categories of mHealth apps: (1) information apps, which provide the public with general health information; (2) diagnostic apps, which are used to input patient information and help guide the physician to a diagnosis; (3) control apps, which allow remote monitoring and control of medical devices such as insulin pumps; and (4) adapter apps, which essentially transform a smartphone into a mobile medical device [6].

Applying these definitions to the FDA guidelines, the agency appears willing to regulate control and adapter apps, which essentially transform mobile platforms into medical devices. The FDA has clarified that it will not regulate informational apps that coach or prompt patients to manage their health or allow them to track their health data [5].

But diagnostic apps have constituted a grey area in the FDA's regulatory purview. In December 2017, the FDA issued a new draft guidance based on the agency's interpretation of the 21st Century Cures Act approved by Congress in 2016. The guidance is intended to inform app developers how it intends to regulate what are called clinical decision support (CDS) apps, which provide diagnostic and treatment recommendations to physicians [7]. CDS apps are distinct and differ from patient decision support (PDS) apps which provide information only to the patient.

The guidance indicates that CDS apps that allow physicians “to independently review the basis for the recommendations” [7] will not be subject to FDA regulation as a medical device. In other words, the FDA said it does not intend to regulate CDS apps that allow the user “to reach the same recommendation on his or her own without relying primarily on the software function” [7].

Examples of apps that may not be covered by FDA regulation under the new guidance include those that provide physicians with recommendations for diagnosing illnesses such as influenza or diabetes mellitus and those that recommend the use of a prescription drug. Also excluded are apps that make chemotherapeutic suggestions to doctors based on a patient's history, test results, and other factors [7].

The FDA’s narrow regulatory framework leaves a large gap for physicians and patients trying to choose an appropriate mHealth app. Attempts to provide clarity have come from state legislation, voluntary certification, patient advocacy groups, professional associations, and commercial services. But the dynamic world of app development so far has defied attempts to regulate or certify these products.

Attempts at voluntary certification for mHealth apps have failed in the past. A group of companies published standards in 2013 for a voluntary app certification program that was to be funded by app developers who paid to have their apps certified [4]. The program was intended to make apps more appealing to customers and to give clinicians greater confidence in recommending them to patients. But the companies suspended the program later that year after it was exposed that two of the certified apps had security problems. The program also suffered from a lack of interest by app developers, of whom only a handful participated [4].

The European regulatory system offers another potential model. In the EU, each member state can file an approval application for a high-risk medical device [8]. The device is then evaluated by a notified body (NB) established within that state and authorized by the state’s public health agency. NBs have the authority to issue the device a Conformité Européenne (CE) mark. This mark denotes conformity with relevant EU requirements for medical devices. A device bearing a CE mark can be sold in any EU member state. Europe has about 76 NBs, which are private for-profit companies that contract with manufactuerers to supply the certifications for a fee [8].

Since the European model was developed to foster commercial policies that encourage trade among member states, not to provide consumer protection, this system has been criticized. For instance, NBs may be reluctant to deny approval of a medical device for fear of losing its client to a competitor [8]. In addition, although the CE mark indicates that the device is in full compliance with European legislation, medical devices approved in Europe need only show safety and performance, but not clinical efficacy [8]. These potential weaknesses have led to criticism that the European NB system values innovation and trade over consumer protection.

## Potential Solution

The US should consider an effective and proven approach that would be analogous the system now used to regulate diagnostic testing. This model, established in 1988 by the Clinical Laboratory Improvement Amendment (CLIA) certifies and ensures the quality of testing in approximately 254,000 US laboratories [9]. Federal agencies rely on nonprofit accrediting agencies with deeming authority under CLIA to ensure that labs comply with federal regulatory standards. Quality standards include staff qualifications, quality control, and recordkeeping. The CLIA approach is unique among federal oversight programs in that it combines oversight with an educational and collaborative approach to ensure quality testing that has allowed tens of thousands of facilities unfamiliar with laboratory technique to comply with federal quality standards. The requirements have been phased in over a period of years to allow greater participation and improvement in the quality and delivery of testing. Virtually all diagnostic testing laboratories comply with CLIA processes today [10].

A similar approach applied to app developers could help ensure that mHealth apps comply with at least basic standards in three areas, namely (1) accessibility, including inclusion of clear language, ease of use, affordability, and usability on all mobile platforms; (2) privacy, including assurances that apps appropriately secure patient records and prohibit data sharing with third parties, such as insurance companies and advertisers; and (3) content, indicating that apps are developed with expert involvement, contain accurate medical information, limit in-app advertising, and reveal both monetization practices, and potential conflicts of interest [2].

The proven CLIA model provides a successful approach to the oversight of mHealth apps and ensures that they are both safe and clinically effective. The science of laboratory medicine has evolved rapidly in the three decades since CLIA was enacted, and the model also has been nimble in responding to new technology and approaches [11].

As CLIA has evolved in the three decades since Congress passed the law, health care providers no longer do business with laboratories that are not CLIA certified. Passage of similar legislation regulating CDS apps may have a similar effect over time, since certification gives the app credibility. Physicians would use or recommend apps preferentially as a result of the certification. Ultimately, accrediting bodies could require it as they now require laboratory certification.

A formal certification process for mHealth apps, particularly those that involve clinical decision making, would give doctors and patients greater confidence in these products as they enter the medical mainstream. Health professionals, patients, and consumer groups must lead the drive for better information, transparency, and usefulness for mHealth apps. As new products enter the realm of diagnosis and treatment, physicians are in a strong position to demand that apps are effective and protect patients, whether used to treat disease or improve health and wellness.

## Abbreviations

CDS: clinical decision support
CE: Conformité Européenne
CLIA: Clinical Laboratory Improvement Amendment
EU: European Union
FDA: Food and Drug Administration
mHealth: mobile health
NB: notified body
PDS: patient decision support
